# Impact of systematic screening for AmpC-hyperproducing *Enterobacterales* intestinal carriage in intensive care unit patients

**DOI:** 10.1186/s13613-020-00754-9

**Published:** 2020-10-29

**Authors:** Elsa Manquat, Matthieu Le Dorze, Gauthier Pean De Ponfilly, Hanaa Benmansour, Rishma Amarsy, Emmanuelle Cambau, Benjamin Soyer, Benjamin Glenn Chousterman, Hervé Jacquier

**Affiliations:** 1 Service de Réanimation Chirurgicale Polyvalente, Département d’Anesthésie Réanimation, Hôpital Lariboisière, AP-HP, 2 Rue Ambroise Paré, 75475 Paris Cedex 10, France; 2 Laboratoire de Bactériologie-Virologie, Hôpital Lariboisière, AP-HP, 2 Rue Ambroise Paré, 75475 Paris Cedex 10, France; 3 Equipe Opérationnelle d’Hygiène, Hôpital Lariboisière, AP-HP, 2 Rue Ambroise Paré, 75475 Paris Cedex 10, France; 4 UMR1137, IAME, University of Paris, Paris, France; 5 UMR-S942, Mascot, University of Paris, Paris, France

**Keywords:** *Enterobacterales*, Extended-spectrum β-lactamase (ESBL-E), High-level expressed AmpC cephalosporinase-producing *Enterobacterales* (HLAC-E), Third-generation cephalosporin-resistant *Enterobacterales* (3GCR-E), Intestinal carriage, Respiratory sample, Intensive care unit

## Abstract

**Background:**

Empirical antimicrobial therapy (EAT) is a challenge for community-acquired, hospital-acquired and ventilator-associated pneumonia, particularly in the context of the increasing occurrence of third-generation cephalosporin-resistant *Enterobacterales* (3GCR-E), including extended-spectrum beta-lactamase *Enterobacterales* (ESBL-E) and high-level expressed AmpC cephalosporinase-producing *Enterobacterales* (HLAC-E). To prevent the overuse of broad-spectrum antimicrobial therapies, such as carbapenems, we assessed the performance of screening for intestinal carriage of HLAC-E in addition to ESBL-E to predict 3GCR-E (ESBL-E and/or HLAC-E) presence or absence in respiratory samples in ICU, and to evaluate its potential impact on carbapenem prescription.

**Materials and methods:**

This monocentric retrospective observational study was performed in a surgical ICU during a 4-year period (January 2013–December 2016). Patients were included if they had a positive culture on a respiratory sample and a previous intestinal carriage screening performed by rectal swabbing within 21 days. Sensitivity, specificity, positive (PPV) and negative (NPV) predictive values and likelihood ratios were calculated for the screening for intestinal carriage of ESBL-E, HLAC-E and 3GCR-E (ESBL-E and/or HLAC-E) as predictor of their absence/presence in respiratory samples. Impact of HLAC-E and ESBL-E reporting on EAT was also studied.

**Results:**

765 respiratory samples, retrieved from 468 patients, were analyzed. ESBL-E prevalence was 23.8% in rectal swab and 4.4% in respiratory samples. HLAC-E prevalence was 9.0% in rectal swabs and 3.7% in respiratory samples. Overall, the 3GCR-E prevalence was 31.8% in rectal swabs and 7.7% in respiratory samples. NPVs were 98.8%, 98.0% and 96.6% for ESBL-E, HLAC-E and 3GCR-E, respectively. Over the study period, empirical antimicrobial therapy was initiated for 315 episodes of respiratory infections: 228/315 (72.4%) were associated with negative intestinal carriage screening for both HLAC-E and ESBL-E, of whom 28/228 (12.3%) were treated with carbapenems. Of 23/315 (7.3%) cases with screening for positive intestinal carriage with HLAC-E alone, 10/23 (43.5%) were treated with carbapenems.

**Conclusion:**

Systematic screening and reporting of HLAC-E in addition to ESBL-E in intestinal carriage screening could help to predict the absence of 3GCR-E in respiratory samples of severe surgical ICU patients. This could improve the appropriateness of EAT in ICU patients with HAP and may prevent the overuse of carbapenems.

## Introduction

Community-acquired, hospital-acquired or ventilator-associated pneumonia (CAP, HAP and VAP) are the most common infections in intensive care units (ICUs) and are associated with high morbidity and mortality rate [1, 2]. Empirical antimicrobial therapy (EAT) is a challenge for ICU physicians. Early antimicrobial therapy is recommended especially in case of sepsis or acute respiratory failure [3] and its appropriateness can only be validated a posteriori when sample cultures and antibiotic susceptibility testing are known. The choice of an EAT is, therefore, difficult and there is a trade-off to find between large-spectrum, which may promote antimicrobial resistances and narrow-spectrum therapy, which may result in treatment inadequacy. Because of frequent long hospital stays, previous antimicrobial exposure, or presence of other individual patient risk factors of multidrug-resistant (MDR) pathogens, broad-spectrum antibiotics are often used, in accordance with the latest recommendations on the management of patients with HAP and VAP [4]. The dramatically increasing occurrence of third-generation cephalosporin-resistant *Enterobacterales (*3GCR-E) leads to increased use of carbapenems [5], with serious adverse effects such as an increase of antibiotic selective pressure [6, 7].

To prevent the overuse of such broad-spectrum antimicrobial therapy, strategies have been developed such as rapid susceptibility testing [8, 9] and intestinal carriage monitoring [10–13]. The 2015 European Society of Clinical Microbiology and Infectious Diseases (ESMID) guidelines provided strong recommendations to implement contact precautions to reduce the spread of beta-lactamase-producing *Enterobacterales* (ESBL-E) in non-epidemic settings [14]. In line with these recommendations, rectal swabs are performed at admission and weekly thereafter in our ICU to screen intestinal carriage of ESBL-E. This approach is included in our institutional infection control policy to implement isolation measures in the identified carrier, and to prevent and identify cross-contaminations.

We have recently shown that in severe ICU patients, negative screening for ESBL-E intestinal carriage could predict the absence of ESBL-E in respiratory samples. This finding was suggested to avoid carbapenem overuse [15].

Most microbiological culture media used to screen ESBL-E are not specific and also detect high-level expressed AmpC cephalosporinase-producing *Enterobacterales* (HLAC-E) [16]. These are classically not reported to clinicians, as there is no recommendation to isolate such patients [14, 17]. Thus, the epidemiology of HLAC-E intestinal carriage, especially in ICU, remains poorly described [18–20] despite its increasing burden [21]. In the present study, we explore the potential clinical relevance of providing information on the intestinal carriage screening for HLAC-E in addition to ESBL-E.

The objectives of this study were (1) to assess the impact of reporting HLAC-E intestinal carriage screening in addition to ESBL-E to predict 3GCR-E (ESBL-E and/or HLAC-E) in respiratory samples in ICU patients with suspected HAP; and (2) to evaluate such strategy on carbapenem prescription.

## Methods

### Study design and inclusion criteria

From January 2013 to December 2016, a retrospective observational study was performed in a 30-bed surgical ICU in a teaching hospital in Paris. Our department is particularly involved in the management of neurological failure, due to the high level of neurosurgery activity in our hospital, which participates in a regional network of stroke centers.

Since rectal swabs and respiratory samples were part of our daily practice and no intervention was tested, the *Ethics Committee* of Société Française d’Anesthésie-Réanimation approved the protocol and waived the requirement of written informed consent. Furthermore, a declaration to *the Commission Nationale de l’Informatique et des Libertés (CNIL)* was done (declaration number: 2214863).

During the study period, all patients with a positive respiratory specimen (endotracheal aspirate, bronchoalveolar lavage, protected distal sampling or sputum) were enrolled. Bacterial documentation, including oropharyngeal flora, was collected and ESBL-E/HLAC-E phenotypes were retrieved (see microbiology section). Respiratory samples were performed in case of pneumonia suspected by the physician in charge, with or without mechanical ventilation. Systemic endotracheal aspirate surveillance culture is not part of our daily practice.

In our institution, screening for ESBL-E intestinal carriage is routinely performed by rectal swabbing within the first 24 h after ICU admission and weekly thereafter. This approach is part of our institutional infection control policy to implement isolation measures in identified carriers.

Respiratory samples with culture results below the diagnostic threshold were excluded (see microbiology section). Redundant respiratory samples, i.e., at least two respiratory samples positive for the same pathogen within 5 days, were excluded. Respiratory specimens without rectal swab cultures within the previous 21 days were excluded. Patients with known intestinal carriage with carbapenemase-producing *Enterobacterales* were excluded.

Clinical characteristics of selected patients were collected to describe the population: age, gender, simplified acute physiology score II (SAPS II), ICU mortality rate, length of stay in ICU, duration of mechanical ventilation and main admission diagnosis.

### Microbiology

#### Screening for ESBL-E and HLAC-E intestinal carriage

Rectal swabs were performed by nurses using ESwab^®^ (COPAN Diagnostics, Italy). Transport medium was then inoculated using PREVI^®^ Isola standardized inoculation system (BioMérieux, Marcy-L’Etoile, France) on selective chromogenic ChromID ^®^ ESBL agar plates (BioMérieux, Marcy-L’Etoile, France). This medium is not specific of ESBL-E [16] and also detects HLAC-E. Growing colonies were identified after a 24-h aerobic incubation at 37 °C using mass spectrometry with MALDI™ Biotyper system (Bruker Daltonics, Germany). Results were expressed qualitatively, and no quantification or semi-quantification of growing colonies has been performed. Antimicrobial susceptibility was tested by the disk diffusion method with Mueller–Hinton agar plates (MH agar plates, BioMérieux, Marcy-L’Etoile, France) according to the EUCAST (European Committee on Antimicrobial Susceptibility Testing) and CA-SFM (Antibiogram Committee of the French Society of Microbiology) guidelines.

3GCR-E isolates showing a synergy zone between expanded-spectrum cephalosporins (ESC) and clavulanate were categorized as ESBL-E, while those without synergy and less than 5-mm increase in the ESC inhibition diameter on cloxacillin-supplemented Mueller–Hinton agar (250 mg/mL) were categorized as HLAC-E.

The following ESC and monobactams were used in our antimicrobial susceptibility testing: cefotaxime, ceftazidime, cefepime, and aztreonam.

Carbapenem-Producing *Enterobacterales* (CPE) were identified using the Xpert^®^ Carba-R Assay (Cepheid, Sunnyvale, CA).

HLAC-E results were not transmitted to clinicians, as there is no recommendation to isolate patients carrying such bacteria, but were prospectively collected in our laboratory information system (Glims, version 8.11.14, MIPS, Gent, Belgium).

3GCR-E were defined as *Enterobacterales* expressing resistance to third-generation cephalosporins, whatever the mechanism (i.e. ESBL-E and/or ESBL).

#### Respiratory samples

Respiratory samples were sputum samples obtained by expectoration after oral care with the assistance of a physiotherapist when necessary, endotracheal aspirates (Unomedical, ConvaTec, Deeside, United Kingdom), bronchoalveolar lavages (BAL) during bronchoscopy by slowly injecting and retrieving from the lung area of interest 100 mL of isotonic saline, and protected distal sampling (Combicath, Plastimed, Le Plessis Bouchard, France) using a fiberoptic bronchoscope. Samples were isolated on agar plates using routine methods according to the French Society of Microbiology guidelines. Microbiological identification and antimicrobial susceptibility testing (AST) were obtained as described above. Respiratory samples were defined as positive when at least 10^7^ CFU/mL were observed in sputum cultures, 10^6^ CFU/mL in endotracheal aspirates, 10^4^ CFU/mL in BAL, and 10^3^ colony-forming units CFU/mL in protected distal sampling. Culture results with microbiological identification and resistance patterns were reported to the physicians within 2 days after sampling.

#### The potential impact of screening for ESBL-E and HLAC-E intestinal carriage on empirical antibiotic therapy

The potential impact of screening for ESBL-E and HLAC-E intestinal carriage on EAT was evaluated for episodes of respiratory infections.

Episodes of respiratory infection were defined as a positive respiratory culture associated with at least 5 consecutive days of antimicrobial therapy. Respiratory colonization was defined for patients with incomplete antimicrobial therapy (i.e., less than 5 days), or who did not receive antimicrobial therapy. Episodes of respiratory infection associated with extrapulmonary infections were excluded. Last, episodes of respiratory infection for which antimicrobial therapy was initiated after the AST result were excluded.

EAT was defined as antimicrobial therapy initiated after respiratory sampling and before culture and AST results. The choice of EAT is routinely guided by local protocol during a daily infectious disease consultation, according to the guidelines [4, 22]. EAT is prescribed by the physician in charge of the patient, considering the length of previous hospitalization, intestinal carriage status, presumed infection origin and hemodynamic status. EAT with carbapenem was recommended for patients with ESBL-E intestinal carriage, following international recommendations [23] and the results of our previous works [15].

EAT was compared to the status of intestinal carriage according to the 4 main resistance profiles reported in the intestinal carriage: ESBL-E(-)/HLAC-E(-), ESBL-E(-)/HLAC-E(+), ESBL-E(+)/HLAC-E(-), and ESBL-E(+)/HLAC-E(+). We defined a “Potential Carbapenem overuse” for patients with suspected pneumonia and without ESBL-E intestinal carriage, for which EAT with carbapenem should be carefully justified [15]. “Potential inappropriate EAT” was defined as an EAT which was not effective on the resistance pattern reported in intestinal carriage.

### Statistical analyses

Quantitative variables were described using the median (interquartile range) and categorical variables using the number (percentage). Sensitivity, specificity, positive predictive value (PPV), negative predictive value (NPV) and likelihood ratios (LR) were determined to assess if ESBL-E and HLAC-E intestinal carriage could predict their presence or absence in respiratory samples. All analyses were performed using R, version 3.5.3.

## Results

### Population characteristics

During the study period, 3228 patients were admitted in the ICU and 468 were included in the study. The demographic data of included patients are described in Table 1.

**Table 1:** Demographic data (*n* = 468)

Variable	
Age, year	60 (48–71)
Gender, male *n* (%)	328 (70.1)
SAPS II, points	46 (32–53)
ICU mortality, *n* (%)	89 (19.0)
Length of stay, days	31 (15–48)
Patients under MV, *n* (%)	443 (94.7)
Main admission diagnosis, *n* (%)	
Neurological failure	292 (62.4)
Sepsis or septic shock	169 (36.1)
Respiratory	44 (9.4)
Abdominal	21 (4.5)
Other	31 (6.6)
Other	104 (22.2)

Data are expressed as absolute values (percentage) and median (interquartile range). SAPS II: Simplified Acute Physiology Score II. ICU: Intensive care unit. MV: Mechanical ventilation

### Respiratory samples and rectal swabs

**Fig. 1:**
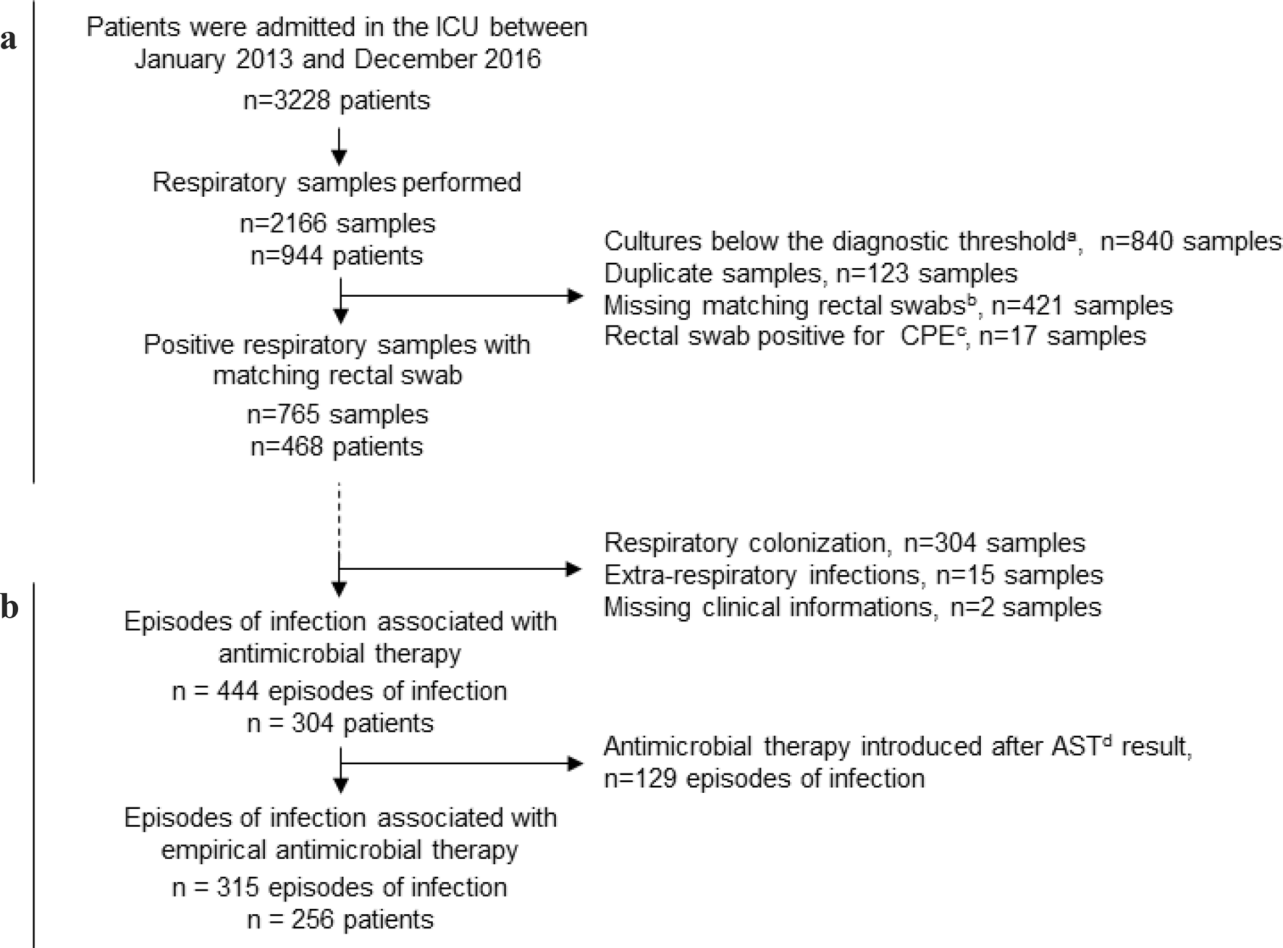
Flow chart of patients included in the study. **a** Data used to test the performances of rectal colonization surveillance to predict presence of HLAC-E, ESBL-E and 3GCR-E in respiratory samples. **b** Data used to test the potential impact of rectal colonization surveillance on empirical antimicrobial therapy of pulmonary infections. ^a^ Respiratory sample was defined as negative when less than 10^3^ colony-forming units (CFU)/mL were observed in protected distal sampling, 10^4^ CFU/mL in BAL, 10^6^ CFU/mL in endotracheal aspirates and 10^7^ CFU/mL in sputum cultures. ^b^ Patients without rectal swab culture available within 21 days before the respiratory sample were excluded. ^c^ CPE, carbapenemase-producing *Enterobacterales.* ^d^ AST: antimicrobial susceptibility testing

A total of 2166 respiratory samples were retrieved from 944 patients. Among them, 1401 respiratory samples were excluded: 840 below threshold samples, 123 duplicate samples for which only one sample was included, and 421 samples with missing rectal swab, 17 were excluded due to carbapenemase-producing *Enterobacterales* intestinal carriage. Finally, 765 respiratory samples were obtained on 468 patients (Fig. 1a). Respiratory samples were mainly endotracheal aspirates (*n *= 476, 62.2%), followed by distal protected aspirates (*n *= 152, 19.9%), BAL (*n *= 81, 10.6%) and sputum cultures (*n *= 56, 7.3%). The median time between respiratory samples and the last positive rectal swab was 6 days (IQR [3–11]) for HLAC-E carriers, and 5 days (IQR [3–8]) for ESBL-E carriers.

### Microbiological epidemiology

Concerning the respiratory samples, *Enterobacterales* (27.7%), *Staphylococcus aureus* (23.8%) and *Pseudomonas aeruginosa* (21.9%) represented the main species identified (Table 2).

**Table 2:** Micro-organism data in respiratory samples

Respiratory samples, *n* (%)	*n* = 765
Oropharyngeal flora	94 (12.3)
Monomicrobial	409 (53.5)
Plurimicrobial	262 (34.2)

Microorganisms in respiratory samples, n (%)	*n* = 988
Gram-positive microorganisms	334 (33.8)
Meticillin-susceptible *Staphylococcus aureus*	235 (23.8)
Meticillin-resistant *Staphylococcus aureus*	3 (< 0.1)
*Streptococcus pneumoniae*	31 (3.1)
*Streptococcus* other than *Streptococcus pneumoniae*	30 (3)
Other Gram positive	35 (3.5)
Gram-negative microorganisms	654 (66.2)
*Enterobacterales*	274 (27.7)
*Escherichia coli*	67 (6.8)
*Klebsiella* spp.	58 (5.9)
*Enterobacter* spp.	53 (5.4)
*Proteus* spp.	31 (3.1)
*Serratia* spp	28 (2.8)
*Citrobacter* spp.	22 (2.2)
Other *Enterobacterales*	15 (1.5)
*Haemophilus* spp.	85 (8.6)
Gram-Negative non-fermenting bacilli	264 (26.7)
*Pseudomonas aeruginosa*	216 (21.9)
*Stenotrophomonas maltophilia*	29 (2.9)
*Acinetobacter* spp.	19 (1.9)
Others Gram negative	31 (3.1)

Data are expressed as absolute values (percentage)

ESBL-E prevalence was 23.8% (95% CI [20.8–26.8%]) in intestinal carriage screening and 4.4% (95% CI [3.0–5.9%]) in respiratory samples. HLAC-E prevalence was 9.0% (95% CI [7.0–11.0%]) in intestinal carriage screening and 3.7% (95% CI [2.3–5.0%]) in respiratory samples. Overall, 3GCR-E prevalence was 31.8% (95% CI [28.5–35.1%]) in intestinal carriage screening and 7.7% (95% CI [5.8–9.6%]) in respiratory samples (Additional file 1).

### Performance of screening for 3GCR-E intestinal carriage as a predictor of 3GCR-E in respiratory samples

Table 3 summarized the sensitivity, specificity, predictive values and likelihood ratios of ESBL-E, HLAC-E and 3GCR-E (ESBL-E and/or HLAC-E) intestinal carriage as a predictor of ESBL-E, HLAC-E and 3GCR-E (ESBL-E and/or HLAC-E) presence or absence in respiratory samples. NPV for ESBL-E, HLAC-E and 3GCR-E intestinal carriage as predictor of their absence in respiratory samples were 98.8% (95% CI [98–99.6%]), 98.0% (95% CI [97.0–99.0%]), and 96.6% (95% CI [95.3–97.8%]), respectively.

**Table 3:** Performance of screening for ESBL-E, HLAC-E and 3GCR-E (ESBL-E and/or HLAC-E) intestinal carriage as predictor of their absence/presence in respiratory samples

	Intestinal carriage screening ESBL-E (+)	Intestinal carriage screening HLAC-E (+)	Intestinal carriage screening 3GCR-E (+)
Sensitivity (%) [95% CI]	79.4% [76.5–82.3%]	50.0% [46.5–53.5%]	69.5% [66.2–72.8]
Specificity (%) [95% CI]	78.8% [75.9–81.7%]	92.5% [87.7–97.4%]	71.4% [68.1–74.7]
PPV (%) [95% CI]	14.8% [14.1–15.6%]	20.3% [19.3–21.3%]	16.9% [15.6–18.2]
NPV (%) [95% CI]	98.8% [98–99.6%]	98.0% [97.0–99.0%]	96.6% [95.3–97.8]
Positive LR [95% CI]	3.7 [2.4–5.1]	6.7 [4.9-8.5]	2.4 [1.3–3.5]
Negative LR [95% CI]	0.3 [0.0–0.6]	0.5 [0.0–1.1]	0.4 [0–0.9]

LR: likelihood ratio. Sensitivity, specificity, positive predictive value, negative predictive values are expressed as percentage [95% CI]. Likelihood ratios are expressed as absolute value [95% CI]. ESBL-E, Extended-spectrum beta-lactamase producing *Enterobacterales*; HLAC-E, high-level expressed AmpC cephalosporinase-producing *Enterobacterales*; 3GCR-E, 3rd generation cephalosporins resistant *Enterobacterales*

### The potential impact of screening for ESBL-E and HLAC-E intestinal carriage on empirical antimicrobial therapy of suspected pulmonary infections

The flow chart of episodes of respiratory infections treated by empirical antimicrobial therapy is described in Fig. 1b. Among 765 positive respiratory samples analyzed, 444/765 (58.0%) were associated with an episode of respiratory infection (i.e., treated by an antimicrobial therapy), 304/765 (39.8%) with respiratory colonization, 15 (1.9%) were excluded because of extra-respiratory infections; and 2 were excluded for insufficient clinical data.

**Fig. 2:**
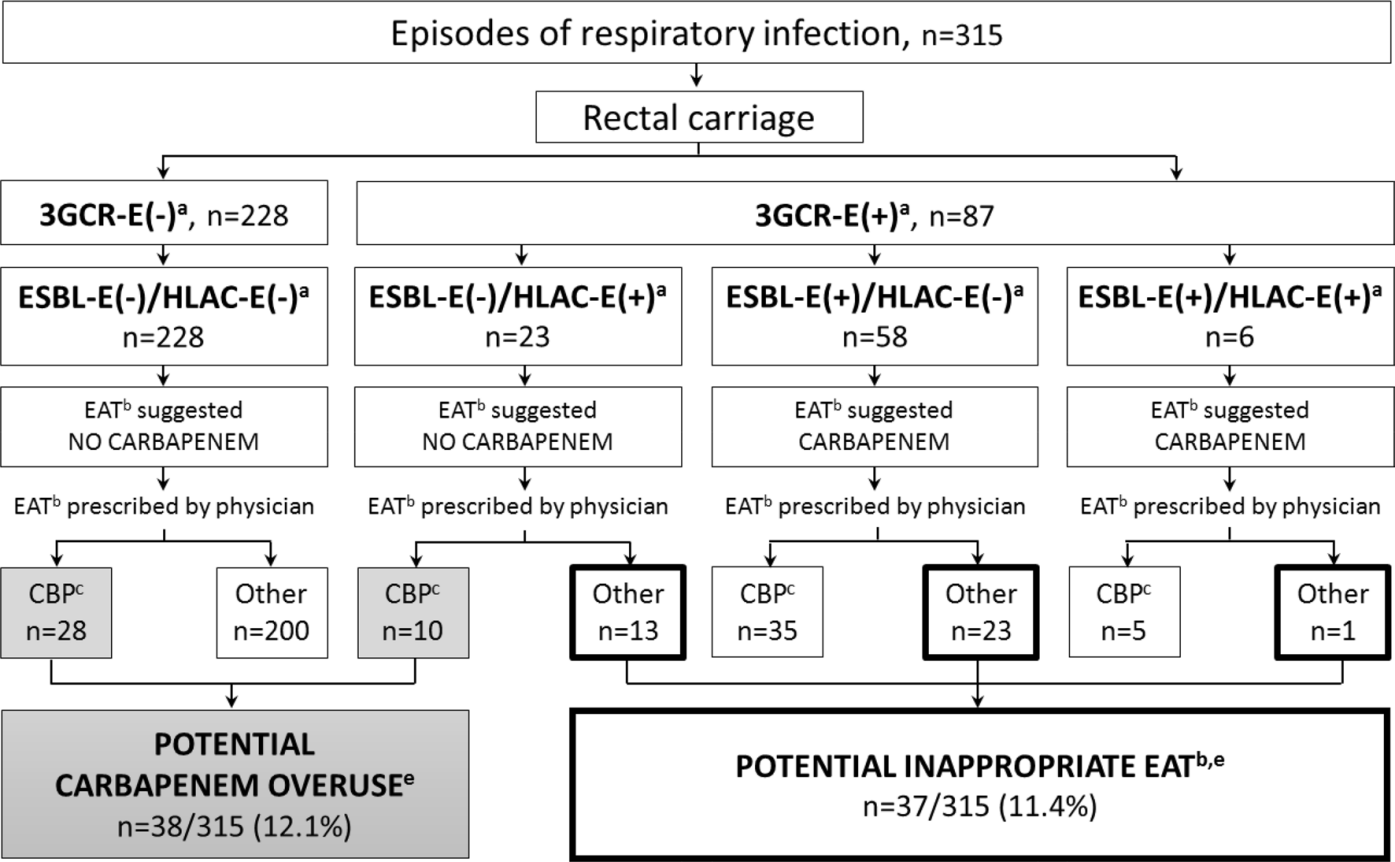
Potential impact of rectal colonization surveillance on empirical antimicrobial therapy (EAT) and on use of carbapenems. ^a^ 3GCR-E: third-generation cephalosporins resistant *Enterobacterales*; ESBL-E: Extended-spectrum beta-lactamase producing *Enterobacterales*; HLAC-E: high-level expressed AmpC cephalosporinase-producing *Enterobacterales*. ^b^ EAT: empirical antimicrobial therapy. ^c^ CBP: carbapenem. ^d^ FEP: céfépime. ^e^ Potential carbapenem overuse and inappropriate EAT were determined by comparing EAT administered to EAT suggested by rectal carriage

A total of 129/444 (29.1%) episodes of respiratory infection were associated with antimicrobial therapy initiated after the AST result, 315/444 (70.9%) with empirical antimicrobial therapy. For these 315 episodes, the results of intestinal carriage were as follows: 228 (72.4%) were ESBL-E(−)/HLAC-E(−), 23 (7.3%) were ESBL-E(−)/HLAC-E(+), 58 (18.4%) were ESBL-E(+)/HLAC-E(−), and 6 (1.9%) were ESBL-E(+)/HLAC-E(+) (Fig. 2).

For 228 and 23 episodes of respiratory infection with ESBL-E(-)/HLAC-E(−) and ESBL-E(−)/HLAC-E(+) intestinal carriage, respectively, 28/228 (12.3%) and 10/23 (43.5%) were treated with carbapenems.

For 64 episodes of respiratory infection with ESBL-E(+)/HLAC-E(−) or ESBL-E(+)/HLAC-E(+) intestinal carriage, 40/64 (65.6%) were treated with carbapenems.

## Discussion

Systematic screening for ESBL-E intestinal carriage by rectal swabbing is recommended in ICU patients in case of recent epidemic or outbreak settings [14, 17] and is commonly used for all patients admitted to ICUs experiencing high rates of intestinal carriage with these pathogens [10, 24, 25]. We recently showed that a negative systematic screening for ESBL-E intestinal carriage was associated with a low risk of ESBL-E in respiratory samples and therefore could help to improve EAT and limit the use of carbapenems [15].

The aim of this study was to explore the clinical relevance of investigating the intestinal carriage of HLAC-E in addition to ESBL-E. To our knowledge, this is the first study exploring the association between intestinal carriage and respiratory presence of HLAC-E in ICU patients.

Microbiological media used to screen ESBL-E are not specific [16] and also detect HLAC-E. Therefore, adding a systematic reporting of HLAC-E presence could be done without additional costs and could have a therapeutic impact.

Our results are in line with recent literature. Here, we confirmed our previous results with good performance of screening for ESBL-E intestinal carriage to predict the absence of ESBL-E in respiratory samples. We reported here a 23.8% prevalence of ESBL-E as compared to 23.2% in Carbonne et al. study and 6.8% observed in the Bruyère et al. study. We report an NPV for the absence of ESBL-E of 98.8% as compared to at least 93.4% and 99.4% in the two previously cited studies, respectively [11, 15].

Regarding the screening for HLAC-E intestinal carriage, we report here a prevalence of 9.0% and an NPV of 98.0% in our population. The prevalence of HLAC-E intestinal carriage remains poorly described [18–20]. The reported prevalence of HLAC-E intestinal carriage in ICU patients was 8.9% and 10.8% for Poignant et al. and Thiébaut et al. studies, respectively, which is in line with our observations [18, 19]. In our study, the rate of HLAC-E respiratory infections in HLAC-E carriers was 20.3% (14/69), a rate close to that reported by Poignant et al. (16.8%) [26]. Thus, negative testing for HLAC-E would be a strong advocate against targeting HLAC-E in the management of suspected HAP. Despite the presence of several MDR pathogens risk factors, as a long length of stay in ICU (median of 31 (15–48) days), and high probability of previous antimicrobial therapy, the NPV of 3GCR-E detection remains high (96.6%).

To go further, we investigated what would have been the clinical impact of combined ESBL-E/HLAC-E intestinal carriage screening on EAT for HAP in our population. We used a strict definition of HAP episodes, i.e., culture above recommended thresholds followed by antimicrobial therapy for more than 5 days. This ensures that the selected episodes were considered as relevant HAP by physicians in charge. Thus, we evaluated the impact of this dual screening for situations in which EAT was deemed necessary. Due to the high NPV of 3GCR-E “Potential Carbapenem overuse”, defined for patients with suspected pneumonia and without ESBL-E intestinal carriage, was identified in more than 10% EAT. Of note, despite recent studies confirmed that cefepime could be used to treat HLAC-E infections [27–29], this was not part, at that time, of our local protocol, and is probably responsible for an overestimation of potential carbapenem overuse. Conversely, “Potential inappropriate EAT”, defined as an EAT which was not effective on the resistance pattern reported in intestinal carriage was identified in more than 10% of EAT.

NPV for 3GCR-E intestinal carriage as a predictor of their absence in respiratory samples was high; therefore, the aim of the second part of our study was to compare prescribed EAT to EAT suggested by rectal carriage, and not to bacterial findings in respiratory samples. Due to the high prevalence of *P. aeruginosa* in our respiratory samples, this approach could be debatable. Therefore, we further explored respiratory samples of patients negative for ESBL intestinal carriage. Of 23 patients with ESBL-E(−)/HLAC-E(+) intestinal carriage, 8 patients had *P. aeruginosa* in the respiratory sample, and all of them were susceptible to ceftazidime or cefepime. Of 228 patients with ESBL-E(−)/HLAC-E(−) intestinal carriage, 40 patients had *P. aeruginosa* in the respiratory sample, and 39/40 were susceptible to ceftazidime or cefepime. This observation is particularly of interest, because cefepime can cover most AmpC-producing and AmpC-hyperproducing Enterobacterales, and is indicated as an EAT for clinically suspected VAP for double antipseudomonal/Gram-Negative coverage [4]. Moreover, in the presence of septic shock, studies have shown that therapy based on antipseudomonal third-generation cephalosporins combined with aminoglycosides is a safe option to improve appropriateness of empiric antimicrobial therapy [30–32]. Therefore, coverage of *Pseudomonas aeruginosa* can be achieved without the prescription of carbapenem, even in case of septic shock.

Our study has some limitations. First, the selective medium we used (ChromID^®^ ESBL) is dedicated to ESBL-E detection, and HLAC-E growth is generally considered as a side effect due to the lack of specificity of such media. This lack of specificity is mainly due to HLAC-E [16], but no studies previously explored the performance of this selective medium for HLAC-E detection.

Second, as detection of HLAC-E in rectal swabs performed weekly is not part of recommendations yet, our population comes from a single center and our results can only be applied to ICUs with similar microbial resistance patterns and prevalence of profile of resistance. However, it should be noted that the incidence of HLAC-E carriage may not have been profoundly influenced by our local ecology. Indeed, regarding HLAC-E acquisition, length of stay and antibiotic-related alteration of the anaerobic flora play a major role compared to cross-transmission [33]. Therefore, HLAC-E carriage and infections depend mostly on the mutant selection from endogenous intestinal microbiota and not on cross-transmission that depends on the bacterial ecology of a given ICU.

Third, the link between intestinal carriage and respiratory samples we evaluate is debatable, since we choose to analyze resistance patterns as a whole, regardless of bacteria species. Especially for HLAC-E (which is most of the time a vertically transferred mutation-promoted overexpression of normally low-level expressed *ampC* gene), knowing that the same species have colonized both intestinal microbiota and respiratory tract would have strengthened the value of intestinal carriage screening to predict the risk of detecting HLAC-E in respiratory samples. In a previous study, Poignant et al. showed that the same species were isolated from the intestinal carriage and clinical samples in all patients experiencing both carriage and HLAC-E infections [18].

Finally, these data were collected retrospectively; the risk of bias is high. However, digitalized laboratory information systems limit this risk. The clinical utility of adding HLAC-E detection and reporting to ESBL-E intestinal carriage screening should be evaluated prospectively, to establish its real impact on empiric antimicrobial therapy, carbapenem overuse, and outcome.

To conclude, we found that the addition of systematic identification and reporting of HLAC-E to current ESBL-E intestinal carriage screening could help to predict the absence of 3GCR-E in respiratory samples of severe surgical ICU patients. This result could help to improve the appropriateness of empirical antimicrobial therapy in ICU patients with suspected HAP.

## Supplementary information

**Additional file 1.** Supplementary table 1a. Prevalence of extended-spectrum beta-lactamase producing Enterobacterales (ESBL-E) in intestinal carriage screening and respiratory samples paired by patients. Supplementary table 1b. Prevalence of Enterobacterales producing AmpC cephalosporinase (HLAC-E) in intestinal respiratory samples paired by patients. Supplementary table 1b. Prevalence of Enterobacterales producing AmpC cephalosporinase (HLAC-E) in intestinal cephalosporins (3GCR-E) in intestinal carriage screening and respiratory samples paired by patients.

## Data Availability

Not applicable.

## Abbreviations

BAL: Bronchoalveolar lavage
CFU: Colony-forming units
EAT: Empiric antimicrobial therapy
ESBL-E: β-lactamase producing *Enterobacterales*
HLAC-E: High-level expressed AmpC cephalosporinase-producing *Enterobacterales*
ICU: Intensive care units
LR: Likelihood ratio
MDR: Multidrug-resistant pathogens
NPV: Negative predictive value
PPV: Positive predictive value
SAPS II: Simplified acute physiology score II
VAP: Ventilator-associated pneumonia
ESC: Expanded-spectrum cephalosporin
