# Correction to: Impact of systematic screening for AmpC-hyperproducing *Enterobacterales* intestinal carriage in intensive care unit patients

**DOI:** 10.1186/s13613-021-00864-y

**Published:** 2021-05-26

**Authors:** Elsa Manquat, Matthieu Le Dorze, Gauthier Pean De Ponflly, Hanaa Benmansour, Rishma Amarsy, Emmanuelle Cambau, Benjamin Soyer, Benjamin Glenn Chousterman, Hervé Jacquier

**Affiliations:** 1 Service de Réanimation Chirurgicale Polyvalente, Département d’Anesthésie Réanimation, Hôpital Lariboisière, AP-HP, 2 Rue Ambroise Paré, 75475 Paris Cedex 10, France; 2 Laboratoire de Bactériologie-Virologie, Hôpital Lariboisière, AP-HP, 2 Rue Ambroise Paré, 75475 Paris Cedex 10, France; 3 Equipe Opérationnelle d’Hygiène, Hôpital Lariboisière, AP-HP, 2 Rue Ambroise Paré, 75475 Paris Cedex 10, France; 4 UMR1137, IAME, University of Paris, Paris, France; 5 UMRS942, Mascot, University of Paris, Paris, France

## Correction to: Ann Intensive Care (2020) 10:149. 10.1186/s13613-020-00754-9

After publication of the original article [1], an error was identified in the authors’ names. The given names and family names were all erroneously transposed.

The correct authors’ names are:

- Elsa: given name
- Manquat: family name
- Matthieu: given name
- Le Dorze: family name
- Gauthier: given name
- Pean De Ponflly: family name
- Hanaa: given name
- Benmansour: family name
- Rishma: given name
- Amarsy: family name
- Emmanuelle: given name
- Cambau: family name
- Benjamin: given name
- Soyer: family name
- Benjamin Glenn: given name
- Chousterman: family name
- Hervé: given name
- Jacquier: family name

The original article has been corrected.
