# Supplementary material for: Impact of systematic screening for AmpC-hyperproducing Enterobacterales intestinal carriage in intensive care unit patients
Source: Ann Intensive Care. 2020 Oct 29;10:149. doi: 10.1186/s13613-020-00754-9 (PMC7594978; doi:10.1186/s13613-020-00754-9)
Supplement: Supplementary file 1 — Additional file 1. Supplementary table 1a. Prevalence of extended-spectrum beta-lactamase producing Enterobacterales (ESBL-E) in intestinal carriage screening and respiratory samples paired by patients. Supplementary table 1b. Prevalence of Enterobacterales producing AmpC cephalosporinase (HLAC-E) in intestinal respiratory samples paired by patients. Supplementary table 1b. Prevalence of Enterobacterales producing AmpC cephalosporinase (HLAC-E) in intestinal cephalosporins (3GCR-E) in intestinal carriage screening and respiratory samples paired by patients. [file 13613_2020_754_MOESM1_ESM.docx]

**Supplementary table 1a. Prevalence of extended-spectrum beta-lactamase producing *Enterobacterales* (ESBL-E) in intestinal carriage screening and respiratory samples paired by patients**

|  | **Respiratory samples ESBL-E (+)** | **Respiratory samples ESBL-E (-)** | **Total** |
| --- | --- | --- | --- |
| **Intestinal carriage screening ESBL-E (+)** | 27 | 155 | 182 (23.8 %) |
| **Intestinal carriage screening ESBL-E (-)** | 7 | 576 | 583 (76.2 %) |
| **Total** | 34 (4.4 %) | 731 (95.6 %) | 765 |

ESBL-E: Extended-spectrum beta-lactamase producing *Enterobacterales*

**Supplementary table 1b. Prevalence of *Enterobacterales* producing AmpC cephalosporinase (HLAC-E) in intestinal carriage screening and respiratory samples paired by patients**

|  | **Respiratory samples HLAC-E (+)** | **Respiratory samples HLAC-E (-)** | **Total** |
| --- | --- | --- | --- |
| **Intestinal carriage screening HLAC-E (+)** | 14 | 55 | 69 (9.0 %) |
| **Intestinal carriage screening HLAC-E (-)** | 14 | 682 | 696 (91.0 %) |
| **Total** | 28 (3.7 %) | 737 (96.3 %) | 765 |

HLAC-E: high-level expressed AmpC cephalosporinase-producing *Enterobacteriaceae*

**Supplementary table 1c. Prevalence of *Enterobacterales* resistant to 3^rd^ generation cephalosporins (3GCR-E) in intestinal carriage screening and respiratory samples paired by patients**

|  | **Respiratory samples 3GCR-E (+)** | **Respiratory samples 3GCR-E (-)** | **Total** |
| --- | --- | --- | --- |
| **Intestinal carriage screening 3GCR-E (+)** | 41 | 202 | 243 (31.8 %)^a^ |
| **Intestinal carriage screening 3GCR-E (-)** | 18 | 504 | 522 (68.2 %) |
| **Total** | 59 (7.7 %)^a^ | 706 (92.3 %) | 765 |

3GCR-E: 3^rd^ generation cephalosporins resistant *Enterobacterales*^a.^ ESBL-E and HLAC-E were both found in 3 respiratory samples and 8 intestinal carriage screenings.
